# Improvement in Glucocorticoid-Induced Osteoporosis on Switching from Bisphosphonates to Once-Weekly Teriparatide: A Randomized Open-Label Trial

**DOI:** 10.3390/jcm12010292

**Published:** 2022-12-30

**Authors:** Toshihiro Nanki, Mai Kawazoe, Kiyoko Uno, Wataru Hirose, Hiroaki Dobashi, Hiroshi Kataoka, Toshihide Mimura, Hiroshi Hagino, Hajime Kono

**Affiliations:** 1 Division of Rheumatology, Department of Internal Medicine, Toho University School of Medicine, Tokyo 143-8541, Japan; 2 Teikyo Academic Research Center, Teikyo University, Tokyo 173-8606, Japan; 3 Hirose Clinic of Rheumatology, Tokorozawa 359-1111, Japan; 4 Division of Hematology, Rheumatology and Respiratory Medicine, Department of Internal Medicine, Faculty of Medicine, Kagawa University, Miki 761-0793, Japan; 5 Department of Rheumatology and Clinical Immunology, Sapporo City General Hospital, Sapporo 060-8604, Japan; 6 Department of Rheumatology and Applied Immunology, Faculty of Medicine, Saitama Medical University, Moroyama 350-0495, Japan; 7 School of Health Science, Faculty of Medicine, Tottori University, Yonago 683-8504, Japan; 8 Department of Internal Medicine, Teikyo University School of Medicine, Tokyo 173-8606, Japan

**Keywords:** bone mineral density, bisphosphonates, glucocorticoid-induced osteoporosis, fractures, teriparatide

## Abstract

This randomized, open-label, multicenter, parallel study imitating real-world clinical practice assessed the effect of switching to weekly teriparatide in patients with glucocorticoid-induced osteoporosis (GIO) with a lumbar spine/proximal femur bone mineral density (BMD) T-score ≤ −2.0 or ≤−1.0 and a fragility fracture. Forty-four patients were randomized. The mean durations of the corticosteroid and bisphosphonate administrations were 90.0 and 51.3 months. The baseline BMD at L1–L4 was 0.828 and 0.826 g/cm^2^ in Groups B (bisphosphonate) and T (teriparatide); at the femur (total), these values were 0.689 and 0.661 g/cm^2^. The mean change in BMD was numerically higher with teriparatide vs. bisphosphonate but not statistically significant. The mean percentage changes from baseline in BMD at L1–L4 after a 72-week treatment were 0.5% and 4.1% in Groups B and T. The incidence of new fractures was higher in the patients taking bisphosphonates vs. those receiving once-weekly teriparatide at 72 weeks (18.2% vs. 11.8%) and 144 weeks (22.7% vs. 17.6%). The mean percentage change in femur (trochanter) BMD (0.035 [0.007–0.063]; *p* = 0.02) was significantly greater with teriparatide vs. bisphosphonates. Adverse events (AEs) were more frequent with teriparatide vs. bisphosphonates. Switching to once-weekly teriparatide tended to increase lumbar spine BMD and reduce the occurrence of new fractures vs. bisphosphonates.

## 1. Introduction

Oral glucocorticoids are widely used for their anti-inflammatory, immunosuppressive, anti-proliferative, and vasoconstrictive effects [1]. Globally, the prevalence of oral glucocorticoid use has been found to vary between 0.5% of the total adult population to nearly 17%, with increasing prevalence among persons over 70 years [2,3,4,5].

A significant negative impact on the bone, such as an increased risk of fracture and trabecular bone loss, has been observed with even slightly elevated circulating levels of glucocorticoids [6] as well as with prior and current exposure to glucocorticoids [7]. Asian studies have reported an increased risk of vertebral fractures with higher doses and a longer duration of the administration of oral glucocorticoids, particularly among patients with rheumatoid arthritis [8,9,10].

Glucocorticoid-induced osteoporosis (GIO), the leading cause of secondary osteoporosis among young adults and the most frequent cause of iatrogenic osteoporosis, is characterized by rapid bone loss upon the introduction of glucocorticoid (first months to a year) due to increased bone resorption, with a subsequent slow progression due to the impairment of bone formation [7], as well as vertebral fractures and an increased risk of hip and other non-vertebral fractures [11]. Patients taking systemic glucocorticoids for the immunosuppressive treatment of various oncologic diseases, rheumatoid conditions, and irritable bowel disease, or those with asthma or chronic obstructive pulmonary disease undergoing long-term treatment with inhaled glucocorticoid formulations, are usually the ones suffering from GIO [1].

The parathyroid hormone (PTH) and its analog, teriparatide (recombinant human PTH [1,2,3,4,5,6,7,8,9,10,11,12,13,14,15,16,17,18,19,20,21,22,23,24,25,26,27,28,29,30,31,32,33,34]), have the potential to improve the skeletal microarchitecture [12], enhance new bone tissue formation, and offer some remediation of the architectural defects in the osteoporotic skeleton [13]. Its preventive effect on vertebral fractures, but not non-vertebral fractures, was demonstrated in GIO subjects [14]. Although bisphosphonates are the first-line treatment for GIO, with daily teriparatide as the second option [15], guidelines advocate the daily administration of teriparatide in patients with established GIO who require long-term steroid treatment [12,16,17,18] due to the increased BMD with teriparatide in postmenopausal women with GIO receiving long-term estrogen therapy [19]. While concurrent therapy with bisphosphonates could reduce the anabolic activity of teriparatide and should be avoided, sequential therapy with teriparatide might consolidate the beneficial skeletal effects. However, there is a lack of data concerning sequential therapy following an antiresorptive agent with PTH [12].

Several studies have reported increased BMD in patients receiving teriparatide at a high risk of fracture [12,15,20,21,22]. The study by Sugimoto T et al. noted significant increases in lumbar and femoral neck BMDs even after 72 weeks, with no marked increase in the incidence of fractures, and indicated that an extended teriparatide treatment duration from 72 weeks to 24 months maintained the inhibitory effect towards fractures. However, Sugimoto T et al. included untreated patients who were scheduled to receive de novo, once-weekly teriparatide administrations [23]. The TOWER and TOWER-GO studies were limited in that they targeted patients who had not received bisphosphonates before the study [20,21]. Thus, there is a paucity of real-world data regarding the use of once-weekly teriparatide among patients with GIO previously treated with bisphosphonate [23].

Furthermore, there may be some advantages of once-weekly vs. daily teriparatide injections. Increased cortical porosity is associated with the daily, but not weekly, administration of equivalent doses of teriparatide [24]. Additionally, weekly teriparatide injections are associated with sustained changes in bone turnover markers [25,26]. Daily teriparatide administration has the same effect on GIO, but the impact of a weekly administration on GIO remains unclear. Moreover, there is a shortage of clinical data regarding the alteration of treatment from bisphosphonates to teriparatide. We hypothesized that switching patients with GIO who presented BMD loss under previous treatment with bisphosphonates to weekly teriparatide for 72 weeks would lead to an increased BMD of the lumbar spine and proximal femur and a reduced number of vertebral fractions compared with bisphosphonates. Thus, the current study, designed to imitate real-world clinical practice, aimed to assess the effect of switching to weekly teriparatide on this patient population.

## 2. Materials and Methods

### 2.1. Study Design and Participants

This study was a randomized, open-label, parallel study conducted across 23 centers in Japan. Patients were registered centrally. Enrollment began in August 2016 and concluded in August 2018. Follow-up visits were conducted 24, 48, 72, 108, and 144 weeks after the baseline assessment.

Patients with rheumatic diseases who had been taking prednisolone at a dose of ≥5 mg/day for at least 6 months, who had been receiving bisphosphonates for at least 6 months, who had a lumbar spine or proximal femur BMD T-score ≤ −2.0, or ≤−1.0, and who had a history of fragility fracture while receiving corticosteroids were included. The type of bisphosphonate was not limited. Patients whose lumbar spine BMD analysis was performed for only one vertebra due to a lumbar spine fracture or artificial bone; with contraindications to bisphosphonates or teriparatide; with a history of teriparatide or denosumab administration; with active infection or active malignancy; under 20 years of age; who were pregnant women, lactating women, or patients who wished to become pregnant; who did not consent to participate in this study; or were judged by the attending physician to be inappropriate for participation in this study were excluded.

The study protocol was approved by the Ethics Committee of Teikyo University (15-080), Toho University Omori Medical Center (M17025, M1702517230), and the Certified Clinical Research Review Board of Toho University (THC18003, THU18003). This research study complied with the Ethical Principles for Medical Research Involving Human Subjects stipulated by the Declaration of Helsinki. All patients enrolled in the study provided written informed consent. The study was registered at UMIN Clinical Trials Registry under the unique identifier UMIN000021987 and the Japan Registry for Clinical Trials under the identifier jRCTs031180311.

### 2.2. Intervention

Eligible patients were randomized in a 1:1 fashion to receive either bisphosphonate (Group B) or once-weekly teriparatide (Group T) using the dynamic allocation randomization technique without blocking. The stratification factors for allocation were age (≥65 years vs. <65 years), sex, corticosteroid dose at the start of the study (prednisolone equivalent of ≥15 mg/day vs. <15 mg/day), and BMD score (T-score of ≥−2.5 vs. <−2.5). The physician in charge at each participating study site enrolled the patients and allocated them to the intervention groups. Group B included patients who continued to receive the original bisphosphonate. Group T included patients who discontinued bisphosphonate at the start of the study and received a subcutaneous injection of 56.5 μg of teriparatide once weekly. After 72 weeks, teriparatide was discontinued, and bisphosphonate was restarted and continued until week 144. Patients continued to receive corticosteroids, but the treating physician could adjust the dose according to routine clinical practice. Concomitant use of active vitamin D and calcium preparations was recommended, and the respective dose was adjusted or discontinued based on serum calcium levels. Concomitant use of other drugs to treat osteoporosis was prohibited during the study.

### 2.3. Efficacy Outcomes

The primary efficacy outcome was BMD of the lumbar spine and proximal femur at 72 weeks, measured using dual-energy X-ray absorptiometry. BMD data at each site were corrected as needed based on the measurements of a standard spine phantom to ensure consistency across study sites.

The secondary outcomes included BMD in the lumbar spine (L1–L4) and proximal femur (segments, including the neck, trochanter, and total) at 144 weeks and young adult mean (YAM). The YAM value compares the BMD of a healthy woman aged 20–44 years as 100% with the BMD of the subject and was calculated as follows:

YAM (%) = measured value/BMD of healthy women aged 22–44 × 100.

Additional investigations assessed at baseline and subsequent follow-up visits included examinations regarding serum tartrate-resistant acid phosphatase 5b (TRACP 5b), serum osteocalcin, and urinary cross-linked N-terminal telopeptide of type I collagen (NTx).

### 2.4. Analytical Populations

The pre-planned analytical populations included (1) the per-protocol (PPS) (target) population, which was defined as the proportion of cases in which (during the 72-week observation) efficacy values existed at least at two of four observation time points, namely, at the start of treatment, 24 weeks, 48 weeks, and 72 weeks, and included patients that conformed to the clinical study protocol and were eligible for analysis (19 cases in Group B and 7 cases in Group T at 72 weeks; 14 cases in Group B and 7 cases in Group T at 144 weeks), and (2) the intention-to-treat (ITT) population, which excluded patients who withdrew consent before 72 weeks of treatment, died, or were transferred to another hospital, and included 20 cases in Group B and 15 cases in Group T at 72 weeks, and 15 cases in Group B and 15 cases in Group T at 144 weeks.

### 2.5. Statistical Analysis

The baseline characteristics of the patients based on safety analysis target population (SAF) were presented as the mean and SD values for continuous variables and the incidence and frequency of categorical variables. The significant differences between group B and group T were tested by Fisher’s exact test for categorical variables or Mann–Whitney U test for continuous variables.

The primary endpoint was the mean percentage change in BMD of the lumbar spine (L1–L4) and proximal femur (total) after 72 weeks of treatment in each group, which was analyzed based on PPS.

The secondary endpoints were mean percentage change in BMD of the lumbar spine (L1–L4) and proximal femur (total) after 72 and 144 weeks of treatment in each group and YAM, which were analyzed based on both ITT and PPS. Statistics—such as mean and SD—relevant to the primary endpoints and secondary endpoints were calculated. Mann–Whitney U test was used to compare the difference between groups B and T.

Multivariate analysis was performed with a linear mixed model (LMM) based on ITT to assess the association between the mean percentage change in the BMD of the lumbar spine (L1–L4) and proximal femur (total) and the variables. Patient’s ID was used as a random effect, and observation time points (24, 48, and 72 weeks), sex, age, and treatment group were used as fixed effects. The last observation carried forward was used to complete missing values for LMM analysis in ITT.

These statistical analyses were performed using Statistical Analysis Language R version 3.6.2 (R Core Team 2019, R Foundation for Statistical Computing, Vienna, Austria). A *p* value of <0.05 was considered significant for all tests.

## 3. Results

### 3.1. Study Participants

**Figure 1:**
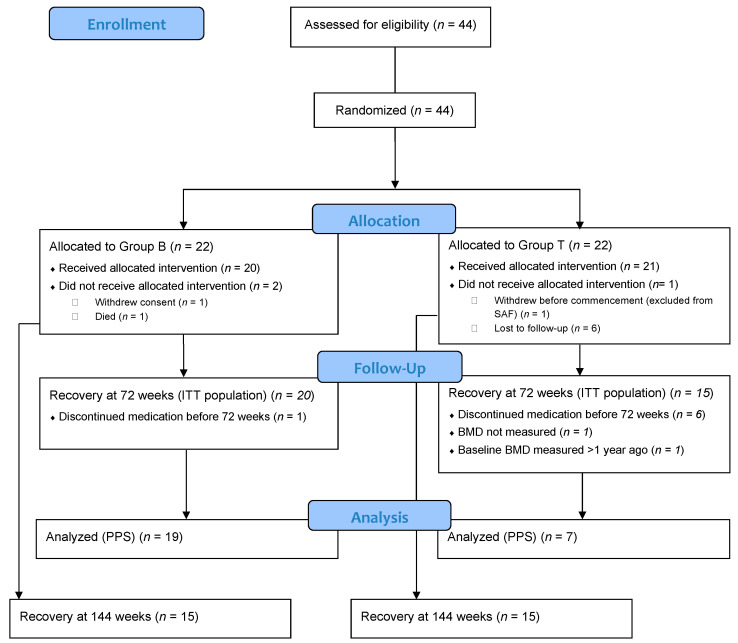
Patient disposition. Group B, bisphosphonate group; Group T, teriparatide group; ITT, intention-to-treat; PPS, per-protocol population; SAF, safety analysis set.

Figure 1 describes the dispositions of the patients and the most common reasons for withdrawal in each group. A total of 44 patients were registered; one patient in Group T withdrew consent before starting the drug administration study and was excluded from all analyses. Therefore, the safety analysis target population (SAF) included 43 patients (22 patients in Group B and 21 in Group T).

The baseline characteristics of the patients are summarized in Table 1. The two groups were generally comparable at baseline. Overall, patients between the ages of 36 and 87 years were enrolled, with a mean age of 68.2 ± 12.6 years, for which 8 were men and 35 were women (of whom 31 were postmenopausal). At the time of enrollment, the corticosteroid dose was similar between the groups (Group B, 5.9 ± 1.5 mg/day; Group T, 7.1 ± 3.7 mg/day). Regarding the overall study population, the mean duration of corticosteroid administration was 90.0 ± 109.2 months, and that for the bisphosphonate administration was 51.3 ± 40.7 months. The BMD at the time of enrollment for the lumbar spine (L1–L4) was 0.828 ± 0.117 g/cm^2^ (YAM, 81 ± 11.776%) in Group B and 0.826 ± 0.12 g/cm^2^ (YAM, 81.143 ± 11.359%) in Group T; for the proximal femur (total), it was 0.689 ± 0.085 g/cm^2^ (YAM, 78.182 ± 10.595%) in Group B and 0.661 ± 0.071 g/cm^2^ (YAM, 75.095 ± 8.062%) in Group T.

### 3.2. Primary Endpoints

**Figure 2:**
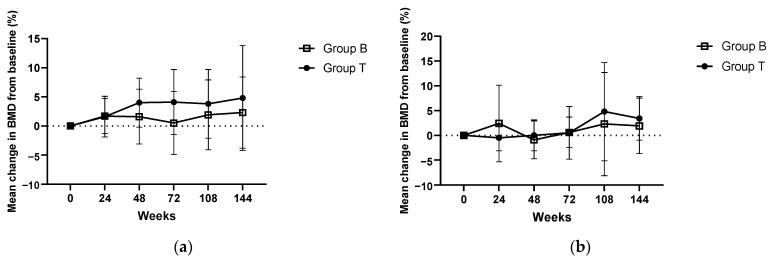
Mean change in BMD for: (**a**) lumbar spine (L1–L4) and (**b**) proximal femur (total) from baseline to 144 weeks for the treatment groups in the PPS. BMD, bone mineral density; Group B, bisphosphonate group; Group T, teriparatide group; PPS, per-protocol population.

Figure 2 illustrates the mean percentage change in the BMD of the lumbar spine and proximal femur (total) from the baseline until the last follow-up visit, i.e., 144 weeks in each group (PPS analysis). Though the mean change in the BMD of the lumbar spine, but not that of the proximal femur, was numerically higher after the teriparatide administration, compared with that after the continuation of bisphosphonates, the difference was not statistically significant over the study period.

The primary endpoint, the mean percentage change in the BMD of the lumbar spine (L1–L4) after 72 weeks of treatment from the baseline in the PPS population, was 0.5 ± 5.4% in Group B and 4.1 ± 5.6% in Group T (*p* = 0.209). Although there was a trend toward a higher BMD in Group T compared to Group B, the difference was not statistically significant. There was no apparent intergroup difference in the mean percentage change in the proximal femur (total) BMD (0.6 ± 3.1%, Group B vs. 0.5 ± 5.3% Group T; *p* = 0.572) at week 72 (Table S1).

### 3.3. Secondary Endpoints

Regarding the secondary endpoints, the mean percentage change in the BMD of the lumbar spine (L1–L4) at 72 weeks after treatment was significantly higher in Group T than in Group B (*p* = 0.014, vs. baseline; Table 2) in the ITT population. In the proximal femur (trochanter), although the difference was not statistically significant, the BMD was numerically higher in Group T. Furthermore, the mean percentage change in BMD at 144 weeks post-treatment was significantly higher in Group T than in Group B with respect to the proximal femur (trochanter) (*p* = 0.038; vs. baseline).

**Figure 3:**
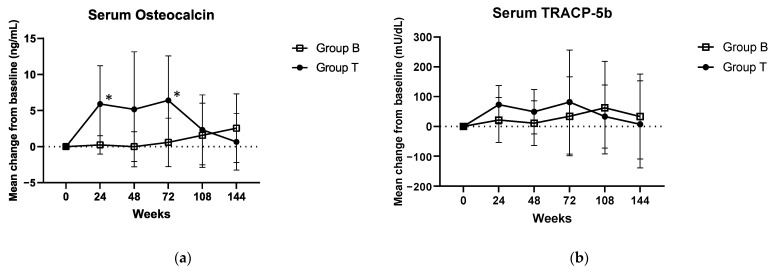

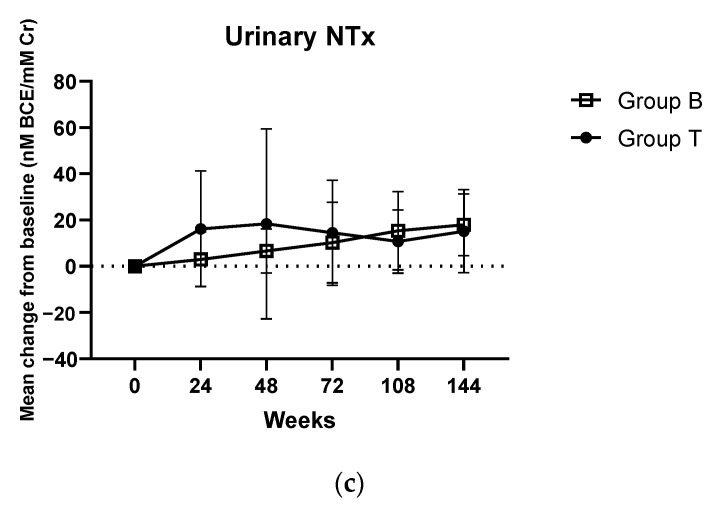
Mean change in (**a**) serum osteocalcin, (**b**) TRACP-5b, and (**c**) urinary NTx over 144 weeks from baseline for the two treatment groups. TRACP 5b, tartrate-resistant acid phosphatase 5b; NTx, urinary cross-linked N-terminal telopeptide of type I collagen. * *p* < 0.05, vs. week 0.

Figure 3 shows the mean change in the biomarkers from the baseline at the defined time points for the two groups. The mean change from the baseline with respect to serum osteocalcin concentration was significantly increased in Group T vs. Group B at 24 weeks (5.9 ± 5.307 ng/mL vs. 0.242 ± 1.27 ng/mL (Group B); *p* = 0.008) and 72 weeks (6.414 ± 6.154 ng/mL vs. 0.584 ± 3.354 ng/mL (Group B); *p* = 0.005). No significant changes were observed regarding serum TRACP-5b and urinary NTx in either group.

Finally, we analyzed the efficacy of the treatment in the ITT population using a linear mixed model. Although the changes in BMD and YAM for both the lumbar spine (L1–L4) and proximal femur (total) were higher in Group T than in Group B, there were no statistically significant differences (Table S2). The mean percentage change in the proximal femur (trochanter) BMD (regression coefficient = 0.035; 95% CI, 0.007 to 0.063; *p* = 0.02) and the mean percentage change in YAM (regression coefficient = 0.046; 95% CI, 0.008 to 0.084; *p* = 0.0230) in Group T were significantly greater than those in Group B.

### 3.4. Adverse Events

All adverse events were recorded up to 144 weeks after treatment, and their causality was assessed. Adverse events were observed in 74% of the patients. Of the total 133 adverse events, 75 occurred in Group B, while 58 occurred in Group T. The total number of serious adverse events that occurred up to Week 144 was 21 (Group B, 15; Group T, 6). The total number of adverse events causally related to each drug was 38 (Group B, 2; Group T, 36) in 11 patients (Group B, 2; Group T, 11), as shown in Table 3. There was a trend toward more adverse events related to teriparatide administration. The adverse events causally related to teriparatide included nausea, headache, dizziness, fever, and fatigue.

### 3.5. New Fractures

The cumulative proportion of new fractures for the treatment groups and overall population were evaluated for the ITT population. At the 72-week follow-up, a total of 15.4% (6/33) of patients were observed to have suffered new fractures, of which 18.2% (4/18) were in Group B and 11.8% (2/15) were in Group T, which increased to a total of 20.5% (8/31) of patients (22.7% [5/17], Group B; 17.6% [3/14]; Group T) at 144 weeks.

A higher incidence of new fractures was observed among the patients receiving bisphosphonates compared to those receiving teriparatide at 72 weeks and 144 weeks. Although there was no significant difference between the groups, the incidence was numerically lower in the teriparatide group.

## 4. Discussion

This first-of-its-kind study was planned in accordance with a real-world scenario, in which many patients are observed to have decreased BMD levels despite bisphosphonate therapy, and it aimed to demonstrate the effectiveness of switching to the weekly teriparatide formulation. In this study, the mean change in the BMD of the lumbar spine, but not that of the proximal femur, was numerically higher after the teriparatide administration compared with that after the continuation of bisphosphonates, but this difference did not reach statistical significance. There was a higher incidence of new fractures in the patients taking bisphosphonates compared with those receiving once-weekly teriparatide at both 72 weeks (18.2% vs. 11.8%) and 144 weeks (22.7% vs. 17.6%). Moreover, the mean percentage change in proximal femur (trochanter) BMD (r, 0.035 [0.007–0.063]; *p* = 0.02) with teriparatide was significantly greater than that observed in the patients who continued taking only bisphosphonates. Thus, we find the present findings to be consistent with the study’s hypothesis.

There is a paucity of existing studies on this topic. Thus, we will discuss the present results in relation to studies with different designs or interventions. Such comparisons should be interpreted carefully. Of note, the most significant difference between the present study and the TOWER [20] and TOWER-GO [21] studies was that patients in these previously conducted studies had not received bisphosphonates prior to the study. This difference in the study selection criteria is of clinical importance as, in real-world practice, many patients are previously treated with bisphosphonates. Nevertheless, the current study’s findings are concordant with the previously reported literature. In the TOWER study, once-weekly teriparatide injections reduced the risk of new vertebral fractures (cumulative incidence, 3.1% with teriparatide vs. 14.5% with placebo [*p* < 0.01]; relative risk 0.20, 95% CI 0.09 to 0.45) and increased BMD by 6.4% in the lumbar spine, 3.0% in the total hip, and 2.3% in the femoral neck (*p* < 0.01) by Week 72 [20]. In contrast, in the TOWER-GO study, BMD was found to have significantly increased by 5.09% from the baseline with teriparatide administration (*p* < 0.05) at 72 weeks [21]. These findings are also supported by those of the Extended Forsteo^®^ Observational Study (ExFOS), which reported a reduced risk of clinical fractures during 24 months of teriparatide treatment, which was maintained 18 months after stopping teriparatide. The adjusted odds of a clinical fracture decreased by 47% in the >12- to 18-month treatment period (*p* = 0.013) compared with the first 6 months, with no statistically significant reduction in the >18- to 24-month interval [29].

While the patients in the present study had previously received bisphosphonates before receiving teriparatide, Sugimoto et al. reported similar findings from the 24-month open-label efficacy research trial examining the BMD in patients with primary osteoporosis and at high fracture risk who received de novo teriparatide once weekly. Compared with the results of the present study, wherein the mean percentage change in the BMD of the lumbar spine (L1–L4, *p* = 0.014) from the baseline was significantly higher in the patients receiving once-weekly teriparatide than in those continued with bisphosphonates after 72 weeks of treatment, Sugimoto et al. found that, compared to the baseline, the lumbar, femoral neck, and total hip BMD increased significantly at Weeks 24, 48, 72, and 104. Significant increases in the lumbar (+1.5%) and femoral neck (+0.8%) BMD were noted at Week 104 compared with Week 72 [23].

In the current study, although the change to the weekly teriparatide formulation increased the BMD in the lumbar spine, there was little change in the proximal femur BMD. This finding was similar to previously conducted studies, which showed a lower rate of BMD increase in postmenopausal patients with osteoporosis who were administered a daily teriparatide formulation along with prior bisphosphonate therapy compared with those who did not receive any bisphosphonates. At 6 months, the authors found an increase in the BMD in the lumbar spine and a decrease in the femur BMD level [30,31]. In this study, the patients with GIO received treatment with the weekly formulation of teriparatide. As expected, until Week 72, there was little change in the proximal femur BMD; however, after 108 weeks, a mild increase in BMD was observed, and thus the effect on the proximal femur was considered weak. A recent head-to-head, open-label comparison study of teriparatide and alendronate administered to Japanese women over 75 years of age with primary osteoporosis found that at 72 weeks of treatment, BMD elevations were significant but similar and discrete in both groups; nevertheless, once-weekly subcutaneous teriparatide injections significantly reduced the incidence of vertebral fractures compared with alendronate [32]. The authors further concluded that these results, along with previous reports [33,34], support the notion that teriparatide’s anti-fracture efficacy is derives primarily from its anti-fracture effects rather than its effects on bone geometry or strength.

Another randomized, double-blind, controlled trial comparing teriparatide (20 μg/day) with alendronate (10 mg/day) with respect to treating GIO in patients at a higher risk of developing fractures noted more significant increases in BMD and fewer new vertebral fractures with teriparatide, and this effect was maintained over 36 months [15]. A single-arm study that assessed the efficacy of a once-weekly teriparatide formulation observed a similar reduction in fracture events and an increase in the lumbar spine BMD in patients with GIO and an inadequate response to bisphosphonates [35]. The present study’s findings are also in concordance with another similar study, wherein adding or switching to teriparatide yielded equal benefits with respect to spine strength in a subgroup of patients who had received prior treatment with alendronate or raloxifene. Though that study had a design similar to that of the current study, it included only postmenopausal women with osteoporosis rather than patients with GIO [36]. The effect of teriparatide has also recently been reported to be maintained after the treatment period. This effect was observed with the daily dosing formulation in patients with GIO who switched to denosumab after 24 months and before bisphosphonate therapy, who were followed up to 48 months [37].

Regarding the biomarker levels assessed, the significant increases in serum osteocalcin levels observed in the present study could be explained by the pharmacological effect of PTH generally seen after 72 weeks of teriparatide administration. This can be confirmed by the fact that these levels were also reduced via bisphosphonates by Week 144. This finding suggests that serum osteocalcin may be a marker of treatment success with respect to teriparatide. In clinical practice, once-weekly teriparatide administration may be effective in patients with BMD loss, despite the widespread use of bisphosphonates along with corticosteroids.

The limitations of the present study include its small sample size and limited generalizability to Japanese patients. The intended sample size was not reached during the enrollment period, which could be related to the difficulty of continuing weekly visits and patient concerns related to the high cost of the teriparatide treatment and potential adverse events. Cases of consent withdrawal and medication discontinuation continued during the observation period, especially in patients receiving teriparatide. This finding could also be related to difficulties in attending follow-up visits and the occurrence of adverse events. There is a need to verify the efficacy of teriparatide on a larger scale in the future.

## 5. Conclusions

This open-label, randomized study showed that switching from bisphosphonates to once-weekly teriparatide increased the lumbar spine BMD and reduced new fractures compared with continue bisphosphonate. Although the difference in the lumbar spine BMD and the incidence of new fractures did not reach statistical significance, these results suggest that once-weekly teriparatide may be effective for the treatment of GIO.

## Figures and Tables

**Table 1:** Patients’ characteristics at baseline.

	All Patients (*n* = 43)	Group B (*n* = 22)	Group T (*n* = 21)	*p* Value
Mean age, in years	68.2 ± 12.6 (36–87)	67.0 ± 13.7 (36–85)	69.5 ± 11.5 (46–87)	0.567
Sex, M:F (with menstruation):F (after menopause)	8:4:31	4:3:15	4:1:16	0.699
Mean corticosteroid dose at enrollment (prednisolone equivalent), in mg/day	6.5 ± 2.8 (5–20)	5.9 ± 1.5 (5–10)	7.1 ± 3.7 (5–20)	0.550
Mean duration of corticosteroid administration to date, in months	90.0 ± 109.2 (7–516)	95.9 ± 103.1 (7–384)	83.8 ± 117.4 (9–516)	0.402
Mean duration of bisphosphonate administration to date, in months	51.3 ± 40.7 (6–144)	57.9 ± 43.2 (6–144)	44.3 ± 37.7 (6–120)	0.291
Frequency of prior fragility fractures after previous steroid initiation	22/43 (51.2%)	11/22 (50%)	11/22 (52.4%)	1.000
Mean lumbar spine (L1–L4) BMD, in g/cm^2^	0.827 ± 0.117	0.828 ± 0.117	0.826 ± 0.12	0.938
Mean lumbar spine (L1–L4) BMD YAM, in %	81.07 ± 11.436	81 ± 11.776	81.143 ± 11.359	0.890
Proximal femur (total) BMD BMD, in g/cm^2^	0.676 ± 0.079	0.689 ± 0.085	0.661 ± 0.071	0.444
Proximal femur (total) BMD YAM, in %	76.674 ± 9.461	78.182 ± 10.595	75.095 ± 8.062	0.331
Mean serum osteocalcin, in ng/mL	8.124 ± 3.926	7.9 ± 3.144	8.37 ± 4.713	0.596
Mean serum TRACP-5b, in mU/dL	242.86 ± 106.902	246 ± 93.836	239.57 ± 121.373	0.985
Mean urinary NTx (corrected for CRE), in mmolBCE/mmolCr	18.98 ± 9.489	18.055 ± 8.382	19.95 ± 10.647	0.732

Data in the table are presented as mean ± SD. BMD—bone mineral density, CRE—creatinine, TRACP 5b—tartrate-resistant acid phosphatase 5b, and YAM—young adult mean.

**Table 2:** Comparison of secondary endpoints in Group B and Group T at 72 and 144 weeks from baseline (intention-to-treat population).

Rate of Change	Week 72			Week 144		
	Group B (*n* = 20)	Group T (*n* = 15)	*p* Value	Group B (*n* = 15)	Group T (*n* = 15)	*p* Value
Lumbar spine (L1–L4)						
BMD	0.8 ± 5.3	4.7 ± 4.5	0.0143	2.5 ± 6.0	8.0 ± 10.9	0.126
YAM	0.5 ± 5.5	4.7 ± 4.5	0.0073	2.6 ± 6.4	8.3 ± 11.0	0.126
Proximal femur (neck)						
BMD	1.3 ± 5.2	1.5 ± 5.1	0.8051	2.7 ± 6.3	3.1 ± 5.6	0.903
YAM	0.9 ± 5.5	1.5 ± 4.9	0.6988	2.6 ± 6.5	3.1 ± 5.6	0.927
Proximal femur (trochanter)						
BMD ^a^	−0.9 ± 3.0	0.9 ± 4.9	0.3426	−1.6 ± 4.2	3.2 ± 6.0	0.038
YAM ^a^	−1.1 ± 3.3	0.7 ± 5.2	0.394	−1.0 ± 3.9	3.0 ± 6.2	0.055
Proximal femur (total)						
BMD	0.5 ± 3.1	1.0 ± 4.2	0.5644	1.5 ± 5.6	2.4 ± 4.4	0.389
YAM	0.3 ± 3.4	0.8 ± 4.0	0.519	1.1 ± 5.7	2.3 ± 4.1	0.345

BMD—bone mineral density; YAM—young adult mean. ^a^ Missing data for up to three patients; BMD was calculated in g/cm^2^; YAM was calculated in %.

**Table 3:** Causally related adverse events.

Adverse Event	Total	Group B	Group T
At least one adverse event	11 (26.2)	2 (9.5)	9 (42.9)
Nausea	4 (9.5)	0 (0.0)	4 (19.0)
Headache	3 (7.1)	0 (0.0)	3 (14.3)
Dizziness	3 (7.1)	0 (0.0)	2 (9.5)
Fever	2 (4.8)	0 (0.0)	2 (9.5)
Fatigue	2 (4.8)	0 (0.0)	2 (9.5)
Back pain	1 (2.4)	0 (0.0)	1 (4.8)
Loss of appetite	1 (2.4)	0 (0.0)	1 (4.8)
Facial flushing	1 (2.4)	0 (0.0)	1 (4.8)
Chest pain	1 (2.4)	0 (0.0)	1 (4.8)
Ureteral stone	1 (2.4)	1 (4.8)	0 (0.0)
Jaw pain	1 (2.4)	1 (4.8)	0 (0.0)
Abdominal pain	1 (2.4)	0 (0.0)	1 (4.8)
Hypercalcemia	1 (2.4)	0 (0.0)	1 (4.8)

Data are presented as frequency (%).

## Data Availability

The data supporting this study’s findings can be provided to academic investigators by the authors upon reasonable request.

## Supplementary Materials

The following supporting information can be downloaded at: https://www.mdpi.com/article/10.3390/jcm12010292/s1, Table S1: Comparison of secondary endpoints in Group B and Group T at 72 and 144 weeks (PPS); Table S2: Influencing factors of treatment groups using a linear mixed model (ITT). Text S1: participating investigators.

## References

[1] Chotiyarnwong P., McCloskey E.V. (2020). Pathogenesis of glucocorticoid-induced osteoporosis and options for treatment. Nat. Rev. Endocrinol..

[2] Bénard-Laribière A., Pariente A., Pambrun E., Bégaud B., Fardet L., Noize P. (2017). Prevalence and prescription patterns of oral glucocorticoids in adults: A retrospective cross-sectional and cohort analysis in France. BMJ Open.

[3] Laugesen K., Jørgensen J.O., Petersen I., Sørensen H.T. (2019). Fifteen-year nationwide trends in systemic glucocorticoid drug use in Denmark. Eur. J. Endocrinol..

[4] Silverman S., Curtis J., Saag K., Flahive J., Adachi J., Anderson F., Chapurlat R., Cooper C., Diez-Perez A., Greenspan S. (2015). International management of bone health in glucocorticoid-exposed individuals in the observational GLOW study. Osteopor. Int..

[5] Overman R.A., Yeh J.Y., Deal C.L. (2013). Prevalence of oral glucocorticoid usage in the United States: A general population perspective. Arthritis Care Res..

[6] Hardy R.S., Zhou H., Seibel M.J., Cooper M.S. (2018). Glucocorticoids and bone: Consequences of endogenous and exogenous excess and replacement therapy. Endocr. Rev..

[7] Briot K., Roux C. (2015). Glucocorticoid-induced osteoporosis. RMD Open.

[8] Nam B., Sung Y.K., Choi C.B., Kim T.H., Jun J.B., Bae S.C., Yoo D.H., Cho S.K. (2021). Fracture Risk and its Prevention Patterns in Korean Patients with Polymyalgia Rheumatica: A Retrospective Cohort Study. J. Korean Med. Sci..

[9] Oh T.K., Song I. (2020). Trends in long-term glucocorticoid use and risk of 5-year mortality: A historical cohort study in South Korea. Endocrine.

[10] Kim D., Cho S.K., Park B., Jang E.J., Bae S.C., Sung Y.K. (2018). Glucocorticoids are associated with an increased risk for vertebral fracture in patients with rheumatoid arthritis. J. Rheumatol..

[11] Compston J. (2018). Glucocorticoid-induced osteoporosis: An update. Endocrine.

[12] Hodsman A.B., Bauer D.C., Dempster D.W., Dian L., Hanley D.A., Harris S.T., Kendler D.L., McClung M.R., Miller P.D., Olszynski W.P. (2005). Parathyroid hormone and teriparatide for the treatment of osteoporosis: A review of the evidence and suggested guidelines for its use. Endocr. Rev..

[13] Lindsay R., Krege J.H., Marin F., Jin L., Stepan J.J. (2016). Teriparatide for osteoporosis: Importance of the full course. Osteoporos. Int..

[14] Cho S.K., Sung Y.K. (2021). Update on Glucocorticoid Induced Osteoporosis. Endocrinol. Metabol..

[15] Saag K.G., Shane E., Boonen S., Marín F., Donley D.W., Taylor K.A., Dalsky G.P., Marcus R. (2007). Teriparatide or alendronate in glucocorticoid-induced osteoporosis. N. Engl. J. Med..

[16] Suzuki Y., Nawata H., Soen S., Fujiwara S., Nakayama H., Tanaka I., Ozono K., Sagawa A., Takayanagi R., Tanaka H. (2014). Guidelines on the management and treatment of glucocorticoid-induced osteoporosis of the Japanese Society for Bone and Mineral Research: 2014 update. J. Bone Miner. Metabol..

[17] Buckley L., Guyatt G., Fink H.A., Cannon M., Grossman J., Hansen K.E., Humphrey M.B., Lane N.E., Magrey M., Miller M. (2017). 2017 American College of Rheumatology guideline for the prevention and treatment of glucocorticoid-Induced osteoporosis. Arthritis Rheumatol..

[18] Kobza A.O., Herman D., Papaioannou A., Lau A.N., Adachi J.D. (2021). Understanding and Managing Corticosteroid-Induced Osteoporosis. Open Access Rheumatol..

[19] Lindsay R., Nieves J., Formica C., Henneman E., Woelfert L., Shen V., Dempster D., Cosman F. (1997). Randomised controlled study of effect of parathyroid hormone on vertebral-bone mass and fracture incidence among postmenopausal women on oestrogen with osteoporosis. Lancet.

[20] Nakamura T., Sugimoto T., Nakano T., Kishimoto H., Ito M., Fukunaga M., Hagino H., Sone T., Yoshikawa H., Nishizawa Y. (2012). Randomized Teriparatide [human parathyroid hormone (PTH) 1–34] Once-Weekly Efficacy Research (TOWER) trial for examining the reduction in new vertebral fractures in subjects with primary osteoporosis and high fracture risk. J. Clin. Endocrinol. Metab..

[21] Tanaka I., Tanaka Y., Soen S., Oshima H. (2021). Efficacy of once-weekly teriparatide in patients with glucocorticoid-induced osteoporosis: The TOWER-GO study. J. Bone Miner. Metab..

[22] Tanaka S., Kuroda T., Sugimoto T., Nakamura T., Shiraki M. (2014). Changes in bone mineral density, bone turnover markers, and vertebral fracture risk reduction with once weekly teriparatide. Curr. Med. Res. Opinion.

[23] Sugimoto T., Shiraki M., Fukunaga M., Hagino H., Sone T., Nakano T., Kishimoto H., Ito M., Yoshikawa H., Kishida M. (2017). 24-month open-label teriparatide once-weekly efficacy research trial examining bone mineral density in subjects with primary osteoporosis and high fracture risk. Adv. Ther..

[24] Zebaze R., Takao-Kawabata R., Peng Y., Zadeh A.G., Hirano K., Yamane H., Takakura A., Isogai Y., Ishizuya T., Seeman E. (2017). Increased cortical porosity is associated with daily, not weekly, administration of equivalent doses of teriparatide. Bone.

[25] Sugimoto T., Nakamura T., Nakamura Y., Isogai Y., Shiraki M. (2017). Profile of changes in bone turnover markers during once-weekly teriparatide administration for 24 weeks in postmenopausal women with osteoporosis. Osteoporos. Int..

[26] Takeuchi Y. (2019). How different is the once-weekly teriparatide from the daily one or the same?. Osteoporos. Sarcopenia.

[27] Miyauchi A., Matsumoto T., Sugimoto T., Tsujimoto M., Warner M.R., Nakamura T. (2010). Effects of teriparatide on bone mineral density and bone turnover markers in Japanese subjects with osteoporosis at high risk of fracture in a 24-month clinical study: 12-month, randomized, placebo-controlled, double-blind and 12-month open-label phases. Bone.

[28] Fujita T., Inoue T., Morii H., Morita R., Norimatsu H., Orimo H., Takahashi H.E., Yamamoto K., Fukunaga M. (1999). Effect of an intermittent weekly dose of human parathyroid hormone (1-34) on osteoporosis: A randomized double-masked prospective study using three dose levels. Osteoporos. Int..

[29] Napoli N., Langdahl B., Ljunggren Ö., Lespessailles E., Kapetanos G., Kocjan T., Nikolic T., Eiken P., Petto H., Moll T. (2018). Effects of teriparatide in patients with osteoporosis in clinical practice: 42-month results during and after discontinuation of treatment from the European Extended Forsteo® Observational Study (ExFOS). Calcif. Tissue Int..

[30] Obermayer-Pietsch B.M., Marin F., McCloskey E.V., Hadji P., Farrerons J., Boonen S., Audran M., Barker C., Anastasilakis A.D., Fraser W.D. (2008). Effects of two years of daily teriparatide treatment on BMD in postmenopausal women with severe osteoporosis with and without prior antiresorptive treatment. J. Bone Miner. Res..

[31] Ettinger B., Martin S.J., Crans G., Pavo I. (2004). Differential effects of teriparatide on BMD after treatment with raloxifene or alendronate. J. Bone Miner. Res..

[32] Hagino H., Sugimoto T., Tanaka S., Sasaki K., Sone T., Nakamura T., Soen S., Mori S. (2021). A randomized, controlled trial of once-weekly teriparatide injection versus alendronate in patients at high risk of osteoporotic fracture: Primary results of the Japanese Osteoporosis Intervention Trial-05. Osteoporos. Int..

[33] Chen P., Miller P.D., Delmas P.D., Misurski D.A., Krege J.H. (2006). Change in lumbar spine BMD and vertebral fracture risk reduction in teriparatide-treated postmenopausal women with osteoporosis. J. Bone Miner. Res..

[34] Zanchetta J.R., Bogado C.E., Ferretti J.L., Wang O., Wilson M.G., Sato M., Gaich G.A., Dalsky G.P., Myers S.L. (2003). Effects of teriparatide [recombinant human parathyroid hormone (1-34)] on cortical bone in postmenopausal women with osteoporosis. J. Bone Miner. Res..

[35] Seno T., Yamamoto A., Kukida Y., Hirano A., Kida T., Nakabayashi A., Fujioka K., Nagahara H., Fujii W., Murakami K. (2016). Once-weekly teriparatide improves glucocorticoid-induced osteoporosis in patients with inadequate response to bisphosphonates. SpringerPlus.

[36] Cosman F., Keaveny T.M., Kopperdahl D., Wermers R.A., Wan X., Krohn K.D., Krege H. (2013). Hip and spine strength effects of adding versus switching to teriparatide in postmenopausal women with osteoporosis treated with prior alendronate or raloxifene. J. Bone Miner. Res..

[37] Hirooka Y., Nozaki Y., Okuda S., Sugiyama M., Kinoshita K., Funauchi M., Matsumura I. (2017). Our-Year Teriparatide Followed by Denosumab vs. Continuous Denosumab in Glucocorticoid-Induced Osteoporosis Patients with Prior Bisphosphonate Treatment. Front. Endocrinol..

