# Supplementary material for: Improvement in Glucocorticoid-Induced Osteoporosis on Switching from Bisphosphonates to Once-Weekly Teriparatide: A Randomized Open-Label Trial
Source: J Clin Med. 2022 Dec 30;12(1):292. doi: 10.3390/jcm12010292 (PMC9820936; doi:10.3390/jcm12010292)
Supplement: Supplementary file 1 [file jcm-12-00292-s001.zip › Tables S1 and S2.pdf]

Supplementary material

Table S1. Comparison of secondary endpoints in Group B and Group T at 72 and 144 weeks

(PPS)

	**Week 72**			**Week 144**		
	**Group B (*n* = 19)**	**Group T (*n* = 7)**	***P* value**	**Group B (*n* = 14)**	**Group T (*n* = 7)**	***p* value**
Lumbar spine (L1-L4)						
BMD	0.5 ± 5.4	4.1 ± 5.6	0.2087	2.3 ± 6.1	4.8 ± 9.0	0.488
YAM	0.2 ± 5.6	4.2 ± 5.6	0.1092	2.4 ± 6.6	5.7 ± 9.6	0.488
Proximal femur (neck)						
BMD	1.8 ± 4.9	2.0 ± 4.2	0.7777	3.4 ± 5.9	2.0 ± 5.3	0.585
YAM	1.4 ± 5.2	2.0 ± 3.9	0.7435	3.3 ± 6.1	2.0 ± 5.3	0.623
Proximal femur (trochanter)						
BMD*	-0.7 ± 3.0	-1.0 ± 3.4	0.9713	-1.0 ± 3.8	3.1 ± 6.1	0.208
YAM*	-0.9 ± 3.2	-1.1 ± 3.2	0.9854	-0.2 ± 3.1	2.7 ± 6.0	0.268
Proximal femur (total)						
BMD	0.6 ± 3.1	0.5 ± 5.3	0.5716	1.9 ± 5.6	3.4 ± 4.4	0.400
YAM	0.4 ± 3.5	0 ± 5.1	0.7776	1.4 ± 5.7	0.3 ± 4.1	0.400

BMD—bone mineral density, PPS—Per-protocol population, YAM—young adult mean

BMD was calculated in g/cm^2; YAM was calculated in %.

Table S2. Influencing factors of treatment groups using a linear mixed model (ITT).

Level	Rate of change	Regression coefficient	95% confidence interval	*p* value
Lumbar spine (L1-L4)	BMD	0.017	-0.004–0.038	0.115
	YAM	0.02	-0.001–0.041	0.073
Proximal femur (neck)	BMD	0.015	-0.012–0.043	0.281
	YAM	0.017	-0.011–0.045	0.244
Proximal femur (trochanter)	BMD	0.035	0.007–0.063	0.020
	YAM	0.046	0.008–0.084	0.023
Proximal femur (total)	BMD	0.01	-0.016–0.036	0.445
	YAM	0.014	-0.013–0.04	0.328

Note: Missing values were supplemented by LOCF; BMD calculated in g/cm^2; YAM calculated in %.

BMD—bone mineral density, ITT—intention-to-treat population, YAM—young adult mean
