# Supplementary material for: Improvement in Glucocorticoid-Induced Osteoporosis on Switching from Bisphosphonates to Once-Weekly Teriparatide: A Randomized Open-Label Trial
Source: J Clin Med. 2022 Dec 30;12(1):292. doi: 10.3390/jcm12010292 (PMC9820936; doi:10.3390/jcm12010292)
Supplement: Supplementary file 1 [file jcm-12-00292-s001.zip › Text S1.pdf]

Text S1

PARTICIPATING INVESTIGATORS.

(Presented in alphabetical order by participating center)

Hiramatsu Clinic: Kazuko Hiramatsu; Jujo Takeda Rehabilitation Hospital: Yukiko Komano; Kanon Clinic: Kaori Imai; Kawasaki Municipal Hospital: Yutaka Okano; Miyazaki Zenjinkai Hospital: Toshihiko Hidaka; Saitama Medical University: Hiroshi Kajiyama; Sanuki Municipal Hospital: Nobuki Nanki, Michiaki Tokuda; Setagaya Rheumatology Clinic: Tomohiko Yoshida; Shimokitazawa Hospital: Akihiro Yamaguchi; Soshigaya Okura Clinic: Hisanori Nakayama; Teikyo University Hospital: Yuki Hatanaka, Keita Nishimura, Kurumi Asako, Hirotoshi Kikuchi, Yoshitaka Kimura, Daisuke Tsukui; Toho University: Sei Muraoka, Kaichi Kaneko, Makoto Kaburaki, Satoshi Mizutani; Tokushima University: Jun Kishi; Tokyo Metropolitan Bokutoh Hospital: Kenichi Shimane; Tokyo Metropolitan Tama Medical Center: Shoji Sugii; Tokyo Women's Medical University: Masayoshi Harigai; Yokohama City University: Ryusuke Yoshimi.
